# Notes on Extramural Interment

**Published:** 1855-09

**Authors:** William Pickells


### NOTES ON EXTRAMURAL INTERMENT,

### AND ITS EXTENSION TO THE CITY OF CORK, AND OTHER CITIES OF IRELAND.

By WILLIAM PICKELLS, M.D.

EARLY in the late session of parliament, a bill was introduced by the late Secretary for Ireland, Sir John Young, to amend the laws relating to the burial of the dead in the Dublin metropolis, and other towns and cities corporate in Ireland. The bill having dropped in consequence of the resignation of Sir John Young, was renewed, and introduced by his successor, Mr. Horsman, under the title of Intramural Burial Bill (Ireland). The bill, however, after having passed the second reading in the House of Lords, has been withdrawn for the present, in consequence of, as stated by Mr. Horsman, certain technical difficulties. What led in the first instance to the introduction of the bill, was, "an influential deputation from St. Anne's parish in Dublin having waited on Sir John Young, soliciting him to bring in a bill for Ireland, similar to that existing for England and Scotland, to prevent burials in the churchyards within the city." Much alarm prevailed at the time, in consequence of the occurrence of some cases of cholera in the city and its immediate vicinity. Among the recommendations of the Committee of the Dublin College of Physicians, appointed in 1853, to report on the requisite preventive and remedial measures, in reference to the apprehended arrival of cholera in this country, one was, "to prohibit, so far as the law permitted, burials within the city." The bill of Mr. Horsman, withdrawn, owing to the difficulties stated by him, for the late session, will, it may be hoped, not fail of success in the next; the necessity of a measure of the kind being not less urgent for Cork and other cities, I believe, of Ireland, than for Dublin. A writer, in a late number of the *Dublin University Magazine,* complaining of the various nuisances, with which, as he expresses it, " the soil of our cities is sodden"—commences with as the worst (the monster nuisance, as it were) by saying, "Festering corpses fill the vaults underneath our churches, or lie within a few feet of the surface in the most populous portions of our cities." Two sessions ago, a Bill passed the legislature, entitled "the Cork Improvement Act," from which much good is expected in regard to street and house cleanliness, ventilation, drainage, &c.; but its provisions did not extend to carrying out the object of extramural interment.

During the famine year, 1847, the want of additional cemetery ground was so grievously felt in this city, as to call forth the following remark in the Fever Hospital Report of the year. " So appalling was the mortality from all causes, that the ordinary burial grounds were quite insufficient, a consideration which should impress on the citizens of Cork the necessity of taking measures for providing a cemetery of suitable extent without the suburbs." During that year, and again during the cholera epidemic of 1849, owing to the limited capacity of the burying grounds, the very poor had to be buried by dozens, or more, in different stages of decomposition in pits; one body being piled or superimposed on another.

In a valuable paper, published in a volume of the *Dublin Medical Journal* for 1853, by Dr. Popham, one of the physicians to the North Infirmary of this city, entitled, "Notes on the Climate and Diseases of Cork," among the causes of insalubrity in the city is particularly noticed that of interments. "Another cause," he observes, "promoting an insalubrious state of the city is found in the intra-urban interments. The graveyards of the churches are of very limited space; and, being nearly all situated in the centre of poor and populous parts of the city, they cannot but exercise an injurious influence. One of the chief medical establishments of the city, the North Infirmary, is built on the skirts of a graveyard, and independently of the depressing effects naturally arising from the association of such a place in the imaginations of the sick, a still greater evil arises from the malarious influence resulting from the continual dredging up the unctuous soil for fresh interments. This was found so great a nuisance during the late epidemic, that the Board of Health had to command the graveyard to be closed against new burials. It is to be regretted that the vested rights of individuals, and the want, though urgent, of a suitable cemetery without the city, prevent the redress of this grievance." Of the graveyards alluded to, those situated in the "Flat", as it is called, of the city, as distinguished from the suburbs, are in the midst of very narrow lanes, which from their crowded, pent-up population, and sanitary condition otherwise, have been long noted as prolific sources of fever and other diseases. The first case of cholera which occurred in London during the epidemic of 1848, was stated to have been "in a narrow, dirty lane, on the water side, full of dung-hills and refuse of every sort, and in which was a *bone shed;*" a description also applying in all particulars to one of these lanes (Godsell's-lane), in which appeared several of the first cases of cholera in this city in the first visitation in 1832. Even though not productive of immediately sensible or manifest ill effects, the practice of intramural or intraurban interment cannot fail to be injurious; the constant inhalation of an atmosphere vitiated by the effluvia from so great a number of putrifying dead, containing many of them the seeds of malignant diseases, tending naturally in its consequences to impair the healthy functions of the human constitution and abridge life.

The subject of *extramural interment* early engaged my attention as a member of the medical profession, at a time, indeed, when from the difficulties in the way of success (the opposition, on the one hand, of vested rights, and, on the other, the interference of private feelings, or time-hallowed prejudices), the idea of extramural interment, on a comprehensive scale for all classes, seemed in this country wild and Utopian, if not revolutionary. In 1818, I

published, in one of the Cork newspapers, under the signature of "One of the Physicians to the Dispensary and to the South Fever Asylum" (a temporary fever hospital during the epidemic), a letter calling attention to a practice which existed among the very poor, of exposing dead bodies in coffins in the public streets (even in the most crowded thoroughfares), for the purpose of procuring money from the charitable for their interment; adducing instances, from cases received into the hospital, of the tendency of the practice to communicate febrile or other infection. Animadverting on the sanitary condition of the city, and the great number of burials within it from want of the means of extramural interment, I computed, from certain data, the number of burials within it since the commencement of the epidemic at about 4000; inferring from the contamination of the air by the miasmata, a deleterious effect "in aggravating and protracting the epidemic". The custom in general of burying within cities (inhuming the dead in the midst of the living) was characterised as, "even to the eye of common sense, a solecism monstrous in the annals of civilized society". To obviate the particular nuisance complained of, the letter concluded by recommending the formation without the precincts of the city of a burial ground which should be free to the very poor, and by appealing to the charitable to contribute the necessary funds at the "imploring voice of humanity!" The appearance of the letter is noticed in Tuckey's *Cork Remembrancer*, by the following entry, under date 1818, November 3rd: "A letter appeared in the *Southern Reporter* of this date, complaining of it being the practice to expose dead bodies in coffins in the public streets, in order to procure money for their interment."

Within a few years afterwards, the cemetery, known at present by the name of the Matthew Cemetery, after its benevolent founder, the Rev. Mr. Matthew, was established without the south suburbs, for all classes, but free to the very poor. This cemetery during some epidemics, which subsequently visited the city, has been of signal utility. At the time of the appearance of the letter, Mr. Matthew was curate of the South parish, the parish in which the Asylum was situated, and he must have been fully cognizant of the facts and statements put forward in it, impressing the necessity of the proposed burial-ground. The cemetery has, however, for some years past, ceased to be free; a restriction rendered necessary, it seems, by its very crowded state; it having, from the agreeableness of its site (on the site of the botanic garden formerly attached to the Cork Institution), become the favourite burial place of many of the middle and more affluent classes.

A nuisance scarcely less serious, connected with the burial of the dead, which at present exists, is the prolonged retention of the dead previous to interment in the dwellings of the living, arising, as it commonly does, from difficulty or inability in procuring the necessary funds for interment; the inability or destitution being sometimes such, that there are not even the means of buying a coffin. Hence instances have occurred of bodies lying above ground, unburied, a week or more, in the close, ill-ventilated, "airless", it may be almost said, rooms of the very poor, until buried at last in self-defence by the penny contributions of the neighbours. Complaints of parties aggrieved by the neighbourhood of nuisances of this sort occasionally find their way into the public prints, to the scandal of our sanitary police. In an instance which came within my own knowledge, the corpse of a man was allowed to remain unburied an entire week, within which period his wife and daughter and another female, an inmate of the family, were attacked with fever, and were in this state received into hospital.

Within the last year or two, a society, under the direction of a committee of highly influential members of the Sanitary Association, has been established in Dublin, entitled, "Towns Improvement Society, to advance practically the Sanitary Condition of Ireland." Among the professed objects of the society, one is, "to prevent the bodies of the dead from imparting evil to the living, previous to and after interment." With this object, the facts now stated may merit their attention. By the Nuisances Removal and Contagion Prevention Acts, guardians are empowered, through the medium of the relieving officer, upon the receipt by him of a certificate duly signed that the body is a nuisance, or injurious to the public health, to cause it to be removed for interment to the next convenient burying-ground; but the mode of proceeding has been found so tardy and circuitous, as to be nugatory in dealing with the evil. How far the Bill of Sir Benjamin Hall may, if extended to Ireland, tend to correct the evil, will remain to be seen. With regard to coffins; formerly the churchwardens had the power of granting coffins for deceased destitute paupers upon the certificate of a physician, or other respectable householder, that the deceased had died in a state of destitution, a fund for this purpose being regularly assessed in the different parishes; but latterly, under the operation of the Poor-law Act, this mode of relief has ceased, it being presumed, in the eye of the law, that no destitute pauper can be found outside the workhouse.

In the sanitary movement in England, as regards extramural interment, the prolonged retention of the dead in the houses of the living before interment is, so far as may depend on difficulty or delay in procuring the necessary funds for interment, sought to be obviated by (in connexion with extramural interment) the reduction of the funeral expenses for all classes; the present very heavy expenses arising for the most part, it is proved, from the heartless exactions of undertakers, who trade on the feelings of relatives at a time of the utmost trouble and confusion. The last report of the London General Board of Health (that from 1848 to 1854) is explanatory on this subject. A main object appears to be, the lessening of the expenses for the working classes, the abuses of the mode of burial by clubs having been long notorious; as a system which, as exposed in a presentment, a few years since, of the Liverpool grand jury, has sometimes produced acts of the most unnatural infanticide!

In the following practice, which has existed in this city and vicinity for upwards of the last twenty years, there is, I conceive, much danger of imparting evil to the living, not from the retention of the dead previous to interment, in the homes of the living, but from their retention previous to interment in the churchyard, or in the vaults of churches. It consists in temporarily depositing the corpse in a vault, commonly the chancel vault, under a church, during the period conceived necessary (six weeks or upwards,) to render it so putrid by decomposition, as to be utterly unfit for the purposes of the anatomist. Instances have occurred of bodies being allowed to remain in this way unburied for months. The practice originated in the intense alarm felt among all classes, as well in Dublin, as in this city, for some time previous to the passing of the Anatomy Act in 1832.* All ground for alarm on this point should have long since ceased, exhumation for anatomical purposes being now unknown; still the alarm, or rather panic, in a diminished degree exists. In a sanitary sense, the practice is even more objectionable than that of interment in vaults under churches, now prohibited by law, or exploded by public opinion in most countries of Europe. In the one case, the depositing is final and permanent; but the other, being only temporary, it exposes to the danger of infection not only those who enter the vault for the purpose of removing the corpse to the churchyard (the stench being described as sometimes intolerable), but persons who may attend the funeral, or if the churchyard, as sometimes happens, be at a distance in the country, even passengers who may go the same way. The extent to which the practice has been carried may be inferred from the large sums said to have been saved in this way, from time to time, by sextons, from fees; the fee varies from a half guinea to one pound per week; an item, which added to the heavy funeral expenses, constitutes no insignificant tax; so that to this city may be applied with the more propriety an observation by a writer in the *Edinburgh Medical and Surgical Journal* (vol. 62,) that "If in this country, it be difficult and expensive to live, it is certainly not less difficult and expensive to die and be buried."†

Intramural interment in large and populous towns tends to contaminate not only the air, but also, it is proved, the water. Burying

---

* Among devices said to have been resorted to in this city in smuggling and exporting dead bodies, one was, inclosing them in piano-cases, pianos being duty free. Pomet in his *History of Drugs*, informs us, that at a time when the human skull was an article of the materia medica, the ossa triquetra being given powdered in epilepsy, the English druggists supplied themselves and the rest of Europe, by importing skulls from Ireland.

† By a strange but mere blunder, confounding the word chancel with chancery, the practice has been sometimes called by the common people, "putting the body in chancery".

in towns, says Dr. Copland, is injurious to the public health in two ways; in the first place by contaminating the air, in the second, by its effects on the digestive organs, in contaminating the water which permeates that soil. This has been particularly remarked of the water of pump wells in the neighbourhood of burial grounds. Instances are noticed in *Walker's Gatherings from Graveyards*, particularly those of London, and in the first volume of *Paris's Medical Jurisprudence.*

In ordering the graveyard, on the verge of which is situated the North Infirmary of this city, to be closed against new burials during the epidemic of 1849, the Commissioners of Health ascribed to it, besides its malarious influence, as mentioned by Dr. Popham, a deleterious effect in deteriorating the water. The water, being supplied by means of a pump erected on the premises, was liable to be tainted by the filtrations from the mass above. In answer to a communication from the owners of vaults in the southern part of Upper Shandon Churchyard, dated February, 1850, they allege: "The sick, who are least able to bear the influence of deleterious agencies, are exposed to miasmata, and to having the water tainted with the products of putrefaction." The North Infirmary, rebuilt on the former site about twenty years since, was founded in 1720, at a time when the laws of the health of masses, now reduced into regular science under the name of hygiene, were little understood, and less attended to, and, before the discoveries of Black, when chemical science was yet in its infancy. The population of the north suburbs, in which it is situated, nearly equals, according to the last census, the united population of the city and of the south suburbs. This consideration would indicate in the selection of the proper site for a new cemetery, a locality somewhere without the north suburbs, at a distance from town accessible to the poor. An agreeable and airy site, open to all the winds, combined with spaciousness of extent, would doubtless, in many instances, even without the coercion of law, induce a voluntary abandonment of the old overcrowded, fetid city churchyards, saturated with the products of putrefaction. The new cemetery would have an effect in redressing the grievance complained of by Dr. Popham, and abating much of the evil of the proximity of the Infirmary to two graveyards. A circumstance which must have tended to crowd to excess the churchyards of this and other cities of Ireland, was the operation of a penal law, long however, obsolete, which prohibited funerals going to old abbey churchyards. A melancholy reminiscence in our local annals attaches to the graveyard alluded to by the Commissioners, the southern part of Upper Shandon churchyard, formed of late years into a regularly consecrated burial ground, under the name of the New Churchyard, to distinguish it from the northern part, the old churchyard, long one of the principal, or rather the principal, burying ground of the city. In the *Cork Remembrancer*, under date 1740, occurs the following entry: "The summer after the hard frost, there was a large pit dug at the back of the green, in Upper Shandon churchyard, where several hundred indigent persons were buried for want of money to purchase graves for themselves!" In the autumn of that year, the memorable epidemics (typhus fever and dysentery of 1740-41, recorded by O'Connell and Rutty) burst forth in this city, carrying in their course desolation over the island.*

The objections to intramural interment have of late years acquired a fearfully augmented force, from the arrival in these islands in an evil hour of the Asiatic cholera, which from its repeated visitations threatens to become endemic among us. That the emanations from the cholera dead are sometimes contagious, or capable of communicating the specific poison or leaven of the malady to the living has been rendered obvious to common observation in this country, from the great numbers who have been attacked after attending cholera wakes and funerals; numbers so great as not to be accounted for by fright or disgust.

In this point of view the circumstances which ushered in the outbreak of 1837 and 1838 in this city, as detailed in the Cork Fever Hospital Report for 1837, are memorably interesting; interesting, not merely in reference to the dangers of intramural interment, but as bearing on the system of quarantine. "The first cases which presented themselves on the 11th of October (1837), were those of three firemen and a passenger from on board the *Vulture* steamer. The three firemen were received into the Fever Hospital, into a ward set apart from the fever wards; the passenger was taken into the North Infirmary. A black lad had died of malignant cholera on board the ship a few hours after leaving London, in which city or its vicinity there were at the time some cases of Asiatic cholera. The mayor of Falmouth refused to permit the landing of the corpse for the purpose of interment. It was brought to Cork, and conveyed to the bridewell, situated in a central and populous part of the city, and, after an inquest there held, was intered in one of the city churchyards. The corpse had lain under a tarpaulin on the deck, opposite to the scuttle which led to the firemen's quarters, who all lived together. One of the firemen and the passenger died on the day of admission into hospital. The other two recovered. The fourth fireman, alarmed by what occurred on board, sailed back for England on the day following that of his arrival. After an interval of a few days, cases began to pre-

* During these epidemics, a poor idiot, nick-named from her habits "Joan of the Pigs", used to carry the dead on her back, and deposit them in a pit in this green. Before the issuing of parish coffins by the churchwardens, which did not date far back, paupers were taken to be buried in a charity coffin so called, which was rolled on a wheelbarrow to the dwelling of the deceased. This coffin served for hundreds. They were buried gratuitously in a pit or pits in the New Churchyard. Dr. Holland, who has lately returned from Oporto, after having spent some time in that city, informs me that a similar custom in regard to the burial of the poor exists at present in that city, with the difference that the family of the deceased pay a trifle for the use of the coffin.

sent themselves for admission into the Fever Hospital from the city."

The epidemic, after its outbreak, visited almost every town and village in the county; in its progress extending with peculiar severity to Limerick county as well as city, and ultimately extending to the rest of Munster and a part of Connaught.

The terms "intramural" and "extramural" are terms much used as applying to interment within and without cities. The bill lately before Parliament was entitled "Intramural Interment Bill (Ireland)". However allowable may be the use of these terms in a popular sense, they are not in strict propriety of language applicable, few cities being at present walled; and of those that had been walled, the greater number by far having outgrown their former dimensions. Hence, if by the prohibition of intramural interment be meant, as implied by the title of the bill, interment solely in towns, the term "interurban" would be the more strictly appropriate. To confine legislation, however, on this subject to the prohibition of interment in towns, would be to leave unprohibited interment in church vaults in the country, an omission which would be a defect. I have known an instance, in which a respectable individual, from having gone into a vault under a country church, in which the body of a relative had been deposited, was seized with symptoms of fever, and died after some days of malignant typhus. With the understanding that the prohibition of "intramural interment" would extend to interment in church vaults in general, as well in country as in town, the term may be conveniently retained. In walled towns, it having been commonly an object to make the most of the given space, the evil of intramural interment must have been considerably aggravated, the extreme narrowness of the lanes and streets, and the height of the houses, presenting so many obstacles to the escape of the effluvia from the churchyards. A part of this city, which may be called "the old town", consisting of a "main street", intersected on both sides by numerous narrow lanes, most of which were never free from fever, was formerly walled. Some of these lanes, previous to the improvements of late years, were so very narrow, that in traversing them during rainy weather, the dispensary physician could not open his umbrella. One, a mere *cul-de-sac*, still remaining, "Coach and Six Lane", of cholera notoriety in 1832, has been so named ironically or by antiphrasis, as if a coach and six might pass through it. The bill which has been introduced into Parliament by Sir William Somerville, "to encourage the providing of improved dwellings for the labouring classes", will, it may be hoped, if it become law, tend to correct evils of this sort.

Without imputing more to intramural interment as a cause of the awful mortality that occurred than may fairly be its due, a melancholy instance, during the civil wars in this country, of the ill effects of intramural interment *in a walled town during a siege*, may be found in the narrative of the sufferings of the inhabitants of Derry during the siege in 1688-89, as narrated by a contemporary annalist. "There were but two places of burial within the walls. Nine thousand corpses were interred within the walls between the 18th of April and the 1st of August in these receptacles of the dead. They were filled to overflowing: the dead bodies putrefying lay heaped on each other. There was a want of earth or other material to cover the putrefying bodies, and the shells aimed at the living frequently fell among the dead, and made hideous exhumations of lately buried bodies. In this sad state, the practice of burying in the back yards became unavoidable, and, as might be expected from the feelings of human nature, continued after the necessity for it had ceased, many desiring that their remains should be deposited with those they loved and lost during this dreadful siege. The mortality, as might be expected, was, after the relief of the city, very considerable. Not more than eighty of the defenders in this siege were killed in battle, or by the shells thrown into the city, but the loss by famine and disease amounted to the number of 9000 above mentioned!"

The practice of burying the dead in the back yards resorted to by the inhabitants during the siege of Derry, owing to the dire exigency of their position, recalls a practice resorted to by the poor of this place, as a mode of precaution, taken under the influence of the alarm caused by exhumation for anatomical purposes, a system which, had it continued, must have been attended with the worst consequences. It consisted, from the inability of paying for the chancel vault, or providing other means of protecting the dead, in inhuming the body in a back yard, or, in situations which did not admit of this, even under the *floor* of the dwelling, during the time conceived necessary (six weeks or upwards) to render the body unfit for the anatomist, the remains being afterwards disinterred and removed to the churchyard.

Besides its *outré* and revolting character, as glaringly repugnant to the usages and decencies of civilised society, the practice appeared so menacing to the public health that I felt bound to call attention to it in two successive letters, in the *Southern Reporter*, under the signature of "One of the Physicians to the Cork Fever Hospital." One, dated April 22nd, 1830, was addressed to the representatives in parliament for city and county, the other, dated September 7th, 1830, was addressed to the editor. The evil was not confined to this city, but extended for a distance of many miles around, including the town of Cove (now Queenstown), in the harbour of Cork. Towards the end of May 1830, a fever of some extent having broken out in that town excited much alarm. In an admonitory address from the altar by the Roman Catholic Bishop, the late right Rev. Dr. Crotty, the outbreak was attributed (I quote from a number of the *Reporter* of that date) "to the practice of interring the dead in the houses, and afterwards disinterring them." Notwithstanding, however, the strictures of the press, and the admonitions of the clergy, instances continued to recur until the arrival of Asiatic cholera in 1832; when the terror inspired by this new and "swift-winged messenger of fate", and the danger of contracting it, effectually put an end to the nuisance; a visitation, which was in other respects a scourge, having, it may be said, in this instance proved a besom!

In resorting to so desperate and revolting an expedient, the poor were, after all, less to be reprehended than pitied. Ardent affection towards their relatives while living, and veneration for their memory when dead, have been ever marked and amiable characteristics of the Irish; qualities, in the enthusiastic degree in which they exist, probably derived from their Celtic ancestors. We have seen in our time the inhabitants of Parga, a town of Albania in Greece, a race to whom a Celtic origin is attributed by M. Malte Brun, in fleeing to a foreign shore from Turkish oppression, transport with them in ships the bones of their progenitors, to place them beyond the reach of possible insult. "Veneration for the dead", observes M. Malte Brun, "a very natural sentiment, mingled with all religions, but of some it appears to have formed the chief part. This was the case with the Celts, whom for other reasons we number with the polytheists."*

Intramural interment, at least in large and populous towns or cities, has ceased, and been generally discontinued throughout the continent of Europe. From the fact that in some of the states of the American Union hygiene is taught as a part of public education, it may be inferred that, at least in those states, the evil of intramural interment has not been overlooked by the authorities. A circumstance somewhat curious is, that the initial movement in favour of reform of the churchyard and church-vault should have been in Spain, and that so religious a monarch as Charles III should have been the first to carry into effect a measure for burying in cemeteries without the precincts of towns. In that kingdom, where the population is crowded into cities, and where the climate is so warm as to occasion rapid putrefaction, the evil was perhaps more felt, and a remedy was more urgent than in northern countries.

In Spain, according to Hecker, *Epidemics of Middle Ages*, "typhus fever first manifested itself, in 1490, in Granada, where it threatened to destroy the army of Ferdinand, and was very fatal to the Moors." The term still used, as expressive of the peculiar efflorescence, "petechia", is of Spanish origin, derived from the Spanish radical word "peca", signifying a freckle, or similar spot.†

---

* An act, somewhat parallel to that of the inhabitants of Parga, is related by Gibbon of the Saxons, where, speaking of the wars of the German Cæsars, in their unsuccessful endeavours during the middle ages to enslave the kingdom of Italy, he says:—"Whole armies were swept away by the pestilential influence of the climate. The survivors brought back the bones of their princes and nobles after boiling away the flesh. The cauldrons for this purpose were a necessary piece of travelling furniture" (a German, it is subjoined in a foot-note, who was using one for his brother, promised it to another after it had been used by himself). The whole Saxon line, according to an authority cited by Gibbon, were extinguished in Italy.

† The typhus of Hippocrates was of an inflammatory, and probably of a

In these islands, the progress of sanitary improvement, as regards extramural interment, has been signally slow or deficient, having by no means kept pace with that of the nations of the continent. Within the last few years, however, it has received in England and Scotland a considerable impulse, by the passing of the different burial acts, providing for the erection of new cemeteries. The horrors of the London graveyards, against which a crusade has been so long and ably kept up by Mr. Walker and others, will, it is hoped, be at length put an end to, under the operation of the New Burial Act. A year or two since, the present Prime Minister, Lord Palmerston, while Home Secretary, proclaimed the church-yards of London to be "a disgrace to the metropolis of the empire, a pestilence, and a nuisance, to be tolerated no longer"! In Liverpool, during the prevalence of the cholera of 1849, which raged with so great severity in that city, in an official document put forth, and which appeared in the public prints, "the practice of burying a number of persons, in different stages of decomposition, in pits", was complained of, as tending to the spread of the malady. "Burying within the city" was at the same time condemned. The sanitary condition of Liverpool should claim the more strict attention, considering the awful mortality from epidemic disease which has occurred of late years on board emigrant ships, immediately after leaving that port, the great outlet of emigration.*

I know not how the French physician Vic d'Azyr, in a note on his translation (published in 1805) from the Italian of the essay of Pittolini, *On the Dangers of Places of Interment*, has fallen into the egregious error of including "among nations that carried the dead without the precincts of cities the Irish"—the Irish and the Danes (*les Irlandois et les Danois*). What might have misled Vic d'Azyr, was the circumstrance of a municipal regulation existing in the Dublin metropolis, that paupers, buried at the public charge, should be buried outside the city. Mr. Abernethy, in his examination before a Committee of the House of Commons in 1832, in alluding to the regulation, proposed it as an example for imitation to cities in England. "I think", said he, "what is done in Dublin ought to be observed in this country. It is directed that paupers, buried at the public cost, should be buried at a distance from the city." In any plan or arrangement which may be adopted for carrying out,

---

remittent or aguish character, attended, as are often such fevers at the present day, with great heat and pain of the eyes. To this form, probably, refers the passage in Exodus: "And I will also do this unto you, I will appoint over you terror, fever, and the burning ague that shall consume the eyes, and shall cause sorrow of heart." In 1829, a paper was read before the London College of Physicians, the object of which was to prove that the λοιμος, or plague of Athens, was not the plague, but rather resembled the fever of the Mediterranean, or the typhoid yellow fever of Gibraltar and Cadiz.

* The tardiness of British legislation in measures of sanitary improvement, may be exemplified in the window-tax not having been repealed in England till 1851, an impost which taxed health by taxing light and air in the dwellings—taxing the vital air, that "chartered libertine"!

in reference to this city, the object of extramural interment, whether by the local authorities or by private speculation, the consideration of a free burial for the indigent poor will, it is hoped, not be lost sight of. There are at present three or four free burial-grounds in the neighbourhood, within distances of two or more miles; but, in addition to the objection of their crowded or over-crowded state, they are so badly enclosed or secured, as to be accessible to injury from pigs and dogs, facilitated by the, in general, very insufficient depth of the graves of the poor, and the frail construction of their coffins, often "not air or water tight".

In bygone times, when the laws of health were little understood or attended to, how much might have been done by the adoption of active sanitary measures of precaution towards limiting, if not preventing, the ravages of those epidemics, which, in many instances, almost converted cities into deserts, the destruction being proportioned to the populousness of the place! "The thicker the grass", said Attila, in refusing, at the siege of Rome, to allow a part of the population to quit, "the more easily is it mown." "Great cities", said our homely proverb, "great graves!" Vic d'Azyr, in his notes on Pittolini, above alluded to, is anxious to render to the French nation a homage which he conceives is justly its due, in proving by extracts from works written on the subject, that it is to it we owe the first elements of sanitary reform, in regard to extramural interment. Dr. Haguenot, professor of medicine in the University of Montpellier, is, according to him, the first among the moderns who (in 1744) exerted himself with energy against the abuse of burying in churches, introduced as it has been by vanity, bad example, and the relaxation of the ancient discipline of the church; the abuse becoming custom, and custom becoming law! What in the first instance opened the eyes of Dr. Haguenot to the dangers of intramural interment, was an occurrence in the cathedral of Montpellier. "Three men died in a church-vault; a fourth had barely time to escape from certain death, and experienced, for some time after, symptoms which made his friends fear for his life. His clothes and his whole person exhaled during several days a cadaverous odour." M. Maret, doctor of medicine, and Secretary of the Academy of Dijon, developed more and more the dangers, in *Mémoires sur l' Usage, où l'on est enterrer les Morts dans les Eglises, et dans l'Enceinte de Villes*, à Dijon, 1773. He remarks on the ill-construction of churches of his time, as in many instances, from their form and position with regard to winds, impeding free ventilation, and favouring the concentration of noxious vapours; and involving danger, in epidemic seasons, to crowded congregations, from the transpiration of the living and the effluvia from the dead! In laying down a plan of extramural interment for the city of Paris, "penetrated", he says, "by the dangers of interment within the walls of cities"; the author of a small tract, published in 1768, entitled *Petit Traité sur les Sépultures*, has anticipated, it would seem, many of the views and suggestions set forth by Mr. Chadwick, in the proposed "London Metropolitan Interment Bill".

The fact of the vitiation of the water of wells in the neighbourhood of churchyards, appears to have been first noticed, as an additional objection to burying within the precincts of towns, by the author of "Memoires sur les Sépultures hors de Villes, 1774." "The water of a well, situated under the cemetery of St. Louis, at Versailles, could not be used on account of its fœtidity." A fatal catastrophe, mentioned by this author as having occurred in a church vault, conveys a fearful warning.

"The lord of a village, two leagues from Nantes, having died, it was intended to give his coffin a more honourable place by removing several others, and among them that of a kinsman who had died three months before. A stench the most fœtid diffused itself through the church. Fifteen assistants died in a short time after. The four persons who disturbed the coffins sank first, and six clergymen, who were present at the ceremony, barely escaped with their lives."* Fourcroy has found, after a long course of investigation, that the seventh or eighth day after the body is put into the ground is the latest at which it can be said to be whole. At the end of that short interval the process of decomposition commences, and produces in the first instance a fluid, which ultimately increases so as to burst the abdomen and let out a gas that is capable of producing the most mischievous effects, and even death, should a person approach too near the corpse."

A French writer, not noticed by Vic D'Azyr, who has written against intramural interment, is the celebrated Abbé St. Pierre, in his "Studies of Nature". "In the first place", he says, "I would have a rule made that no one should be buried in a church." He sketched a plan of extramural interment for the city of Paris, allotting, in an island of the Seine, a distinct receptacle for the ashes of such as may have deserved well of their country, not less by the arts of peace than by achievements in war. In regard to the abuse of burying in churches, "the principal obstacle to this necessary reform does not," he observes, "arise from the clergy, but from the great and the rich, who, seldom disposed to crowd the church in their lifetime, are eager for admission into it after their death, that the people may admire their superb mausoleums, and

* To other facts on record, tending to show that mephitic exhalations are sometimes so dangerous vehicles as to be adequate even to the production of epidemics, or to the envenoming of them when reigning, may be added the following from an interesting work, lately published in this city, by John Lindsay, Esq., entitled "Views of the Parthian History and Coinage." "A pestilence", he says, "which broke out in Babylon in the second century, the Roman writers attributed to a singular cause, to a dreadful stench, which proceeded from a golden coffer in the temple of Apollo (Belus), which was opened by a Roman soldier in search of plunder." Might not this have been sepulchral, inclosing the remains of some distinguished chief or hero? In the Iliad, we read the ashes of Hector were "enclosed in a golden coffer," *ες χρυσειην λαρνακα.*

their virtues as pourtrayed in brass and marble. But thanks to the allegorical representations of our artists, and the Latin inscriptions of our literati, the people know nothing about the matter, and the only reflection they make at the sight of them is, that all this must have cost an immense sum of money, and that such a vast quantity of copper might have been much better employed in making kettles!"*

The great extent to which the practice of burying in churches was carried at the time when St. Pierre wrote, may be inferred from the indignant exclamation of Voltaire—"What! are our temples to be paved with carcases!"

---

### EVERY MAN A LANDED PROPRIETOR.

* Fulsome epitaphs of this sort prompted the lines of Byron:—

"When they are dead, upon their tomb is seen,
Not what they were, but what they should have been."

